# The Hans Kai trial: study protocol of a mixed methods randomized controlled trial evaluating a peer-led health promotion program for adults with or without noncommunicable diseases

**DOI:** 10.1186/s13063-023-07708-z

**Published:** 2023-10-24

**Authors:** Margherita Cameranesi, Rebecca Mollard, Robert Balshaw, Dylan MacKay

**Affiliations:** 1 Department of Food and Human Nutritional Sciences, Faculty of Agriculture and Food Sciences, University of Manitoba, 110 RCFTR 196 Innovation Dr, Winnipeg, MB R3T 2N2 Canada; 2 Chronic Disease Innovation Center, Seven Oaks General Hospital, Winnipeg, MB Canada; 3 George and Fee Yee Centre for Healthcare Innovation, University of Manitoba, Winnipeg, MB Canada

**Keywords:** Health, Health promotion intervention, Mixed methods randomized controlled trial, Protocol, Canada

## Abstract

**Background:**

A significant proportion of Canadian adults is impacted by chronic noncommunicable diseases. These conditions may be improved by peer-led health promotion interventions that target modifiable risk factors; however, to date, there is mixed evidence on the effectiveness of these interventions. Unlike other health promotion programs, Hans Kai is grounded in a holistic model of health that simultaneously addresses multiple determinants of health at different levels of human ecology. In Hans Kai, a set of informational sessions that are delivered in a group setting by healthcare professionals are followed by regular peer-led group meetings in a self-governed support group setting that is designed to promote implementation of newly learned health competences. The Hans Kai trial described here aims to evaluate the efficacy of the Hans Kai program in promoting the health and wellbeing of its participants and investigate the experiences of the Hans Kai participants and facilitators.

**Methods:**

This research will involve a mixed methods trial combining an experimental component with a qualitative component. The experimental component will involve a 6-month 2-group parallel superiority randomized controlled trial (RCT) in which 105 participants will be randomly assigned to two conditions, an intervention group (*n* = 70) that will participate in the Hans Kai program and a control group (*n* = 35) that will have access to standard care using a computer-generated random sequence; blinding will not occur. The RCT will test the impact of the program on several health outcomes and will be followed by a 12–18-month observational follow-up study that will provide data on the long-term durability of the 6-month RCT health outcomes. The qualitative component will investigate the experiences of program participants (*n* = 30) and facilitators (*n* = 15) to identify the main strengths and limitations of Hans Kai, uncover potential implementation issues, and elucidate the mechanisms through which the program works. The population of interest will include adults aged 18 + with or without chronic health conditions who self-report an interest in taking control of their own health and improving their lifestyle. In the RCT, all outcomes of interest will be measured using a multi-method approach, involving self-report questionnaires and objective indicators, and within-subject mean changes in outcomes over time between the two groups will be compared to address the RCT aims. Similarly, in the qualitative component, a multi-method approach, involving in-depth individual interviews, photovoice, and online surveys, will be used to reach a deeper and more nuanced understanding of the program strengths, how the program works, and for which people it is more effective. Adaptable components of the program will also be investigated and modified according to the feedback provided by the RCT participants. In the mixed methods integration of evidence, the qualitative findings will be used to explain the quantitative RCT results.

**Discussion:**

The RCT findings will help support the further development and use of Hans Kai as well as other peer-led health promotion interventions.

**Trial registration:**

United Stated Clinical Trial Registry ClinicalTrials.gov (registration# NCT03949725; Protocol version 2, June 22nd, 2022).

**Supplementary Information:**

The online version contains supplementary material available at 10.1186/s13063-023-07708-z.

## Background

Collectively, chronic diseases—also referred to as noncommunicable diseases—are a growing global epidemic [1]. Chronic diseases, such as cardiovascular diseases, diabetes, obesity, respiratory diseases, arthritis, cancer, and mental health disorders account for the largest causes of preventable death in Canada and the world. These conditions also constitute the largest avoidable burden on the Canadian public healthcare system. The Public Health Agency of Canada estimates that more than 40% of Canadian adults ages 20 + have at least one common noncommunicable disease [2]. These conditions become progressively more common among Canadians of older ages, significantly increasing their mortality and morbidity risk, especially as they age. Most chronic conditions experienced by Canadian adults are linked to modifiable risk factors, including tobacco smoking, physical inactivity, unhealthy eating and sleeping patterns, and alcohol use that may be addressed by health promotion interventions aiming to prevent and control noncommunicable diseases [3].

Peer-led health promotion programs have been used as an intervention to address noncommunicable diseases, as well as social isolation and loneliness in various groups. In peer-led or lay-led health promotion programs, participants are involved in health education activities and form peer groups to provide supportive, preventative, and potentially therapeutic services to one another [4–6]. In these programs, positive health outcomes and overall wellbeing are fostered by leveraging the emotional support that the peer group provides to facilitate the consistent implementation of healthy practices in participants’ everyday lives; they bridge the gap between possessing health knowledge and implementing that health knowledge in practice. Additionally, by providing consistent social support through peer connections, peer-led programs address social isolation and loneliness of program participants, which are both independent risk factors for poor health outcomes in adults, especially as they age [7, 8].

Numerous types of peer-led health promotion programs have been used to promote the health and wellbeing of diverse populations, with or without chronic health conditions [9–12]. Evaluation studies that have assessed the effectiveness of these programs have shown mixed results. For instance, a Cochrane meta-analysis examined lay-led educational programs for the self-management of chronic conditions to determine the effects of these programs on participants’ health status, health-promoting behaviors, healthcare use, and self-efficacy [13]. The meta-analysis focused specifically on programs with peer leaders who had the condition of interest and included a total of 17 studies published between 1986 and 2005. Programs with participants without chronic conditions were excluded. The meta-analysis findings showed that peer-led programs may marginally improve health status in the short term and have a modest effect on health distress. The study also showed that the included programs may lead to improvements in cognitive symptom management and self-efficacy to manage symptoms, increase in frequency of aerobic exercise, and reduction in healthcare utilization.

More recent research evaluating the effectiveness of peer-led health promotion programs have also shown mixed results. In a study published in 2009, 530 university students were randomly assigned to a peer-led HIV/AIDS education program and a similar adult-led program to evaluate their effects on participants’ health-related knowledge [9]. The study showed that the students in the peer-led program gained health-related knowledge after the intervention. However, this did not translate into behavioral changes.

Peer-led programs have been shown to endorse health-promoting behaviors in other settings. For instance, a randomized control trial (RCT) found that, after completing a peer-led health promotion program, adult participants presented lower diabetes risk and better adherence to physical activity, compared to a group of adults who had participated in a similar program led by a professional [14]. Similarly, a study involving 543 adults aged 50 + with at least one risk factor for cardiovascular disease found that adults who participated in a peer-led group-based health promotion intervention had better smoking cessation scores, compared to adults who were assigned to a self-management group [15].

Peer-led programs have also shown promising results in promoting positive mental health. An RCT examining the short-term effects of a peer-led educational program delivered before mental health treatment found that adults assigned to the peer-led group had better self-management knowledge, compared to a control group who received no education [16]. Similarly, another study found that, after completing a peer-led group intervention, adults with diabetes experienced a decrease in depressive symptoms and an increase in self-efficacy [17]. Therefore, although some studies have found peer-led health promotion programs to be effective interventions to promote participants’ mental health and social connections, the effects of these programs on communicable diseases are mixed. It is also unclear what program characteristics are linked to positive outcomes and why. The clinical trial described here was designed to address these knowledge gaps.

This paper describes the protocol of the *Hans Kai trial*, a mixed methods RCT evaluating the Hans Kai program, a community-based peer-led health promotion program for adults with or without chronic health conditions. Since Hans Kai was first implemented in the Canadian context in 2010, a mixed methods convergent parallel study that was participatory in nature and community based was conducted to evaluate the program effectiveness [18]. Quantitative pre- and post-test data were collected using participant surveys, while one-on-one interviews with program participants were used to collect qualitative data. A total of 63 adults participated in this study, although not all study participants completed all data collection activities. The results of this preliminary study showed significant improvements in the mental health of program participants following their participation in Hans Kai. Additionally, 66% of study participants experienced positive behavioral changes (e.g., more physical activity and a healthier dietary pattern) following their participation in the Hans Kai program. Additional positive health-related changes reported by study participants included overcoming social isolation and experiencing enhanced peer support; improving health knowledge and access to health services; perceiving inspiration, motivation, and accountability; and experiencing empowerment due to monitoring one’s own health indicators. Although promising, the results of this preliminary evaluation of the Hans Kai program are limited due to the use of a correlational research design, involving a very limited qualitative component, a small sample, and a narrow focus on only a few health-related outcomes. Additionally, program facilitators were not included in this preliminary evaluation of Hans Kai.

The mixed methods RCT described in this protocol has been designed to address the methodological limitations of the previous study conducted to evaluate the Hans Kai program and provide evidence to clarify the mixed evidence regarding the effectiveness of peer-led health promotion programs. This clinical trial is innovative for several reasons. First, it is the first RCT to be conducted to evaluate the effectiveness of the Hans Kai program in the Canadian context, and therefore, it will either provide robust evidence on the effectiveness of this program in promoting the health and wellbeing of Canadian adults or clarify the reasons behind its ineffectiveness in this context. Additionally, the measurement of numerous wellbeing-related outcomes over time and the mixed methods nature of the trial make this a unique research study. During the clinical trial, numerous wellbeing-related outcomes will be measured to elucidate if the program is more effective in promoting certain areas of wellbeing rather than others. This information, together with the qualitative findings on program participants’ unique experiences in Hans Kai, will be useful in planning targeted enrolment of adults who are impacted by specific life circumstances or chronic conditions to optimize organizational resource use and positive outcomes in future Hans Kai participants. The extensive qualitative component of the trial will include program participants and facilitators and will help unveil the mechanisms through which Hans Kai works in promoting participants’ health and wellbeing. The integrated mixed method findings will help support the further development and use of Hans Kai as well as those of other peer-led health promotion programs in the Canadian context.

### The Hans Kai Program

The concept of Hans Kai or “group learning” originated in Japan, where this approach is used to help people enhance their health knowledge, their ability to monitor their own basic health indicators, and their wellness-focused behaviors [19]. Hans Kai was adapted to the Canadian context and healthcare system by a group of NorWest Co-op Community Health (NorWest; http://norwestcoop.ca) staff members who developed the Hans Kai program through collaborative efforts between healthcare professionals, community members, researchers and representative of the local government, and started offering it in 2010 [18].

This process followed several steps. First, the Hans Kai steering committee was assembled by bringing together 20 Canadian adults from different backgrounds and with unique lived experiences. The advisory committee involved approximately 15 healthcare professionals working at NorWest, including counsellors, health promoters, dieticians, and general practitioners, as well as some researchers affiliated with the University of Manitoba, representatives of the provincial government, and community member representative of the community NorWest serves. Next, the committee identified the major health concerns of community members and analyzed the programs that NorWest was offering at the time to ascertain if any of these programs may be modified to meet the health needs that had emerged. Given that none of the programs NorWest was offering at the time was deemed adequate to meet the health and wellbeing needs of community members, the Hans Kai program was developed to educate community members on health-related issues and potentially prevent the communicable diseases that they were more likely to experience. The Hans Kai health school curriculum was developed through a collaborative effort among all committee members and is being slightly revised constantly according to the ever-changing needs of community members. Community members and healthcare professionals played an especially prominent role in developing the curriculum to make sure that the information delivered during the Hans Kai health school truly reflects the needs of community members and the most current standard of care in the Canadian context. The final step in this process involved informally pilot testing the newly developed program with the first Hans Kai group to revise the program based on participants’ feedback. The main change that was made at this stage was the inclusion of a group leader called “champion” in each Hans Kai group.

Hans Kai is a peer-led, preventative, self-sustaining, community-based health promotion program for adults with or without noncommunicable diseases who wish to maintain or improve their health. Hans Kai empowers individuals to take control of their own health and provides a unique opportunity for participants to have an active role in improving or maintaining their health and wellbeing. The program is a health promotion program for adults of all ages, genders, and socioeconomic circumstances that combines health education with social support in a peer-led, self-sustaining model.

**Fig. 1:**
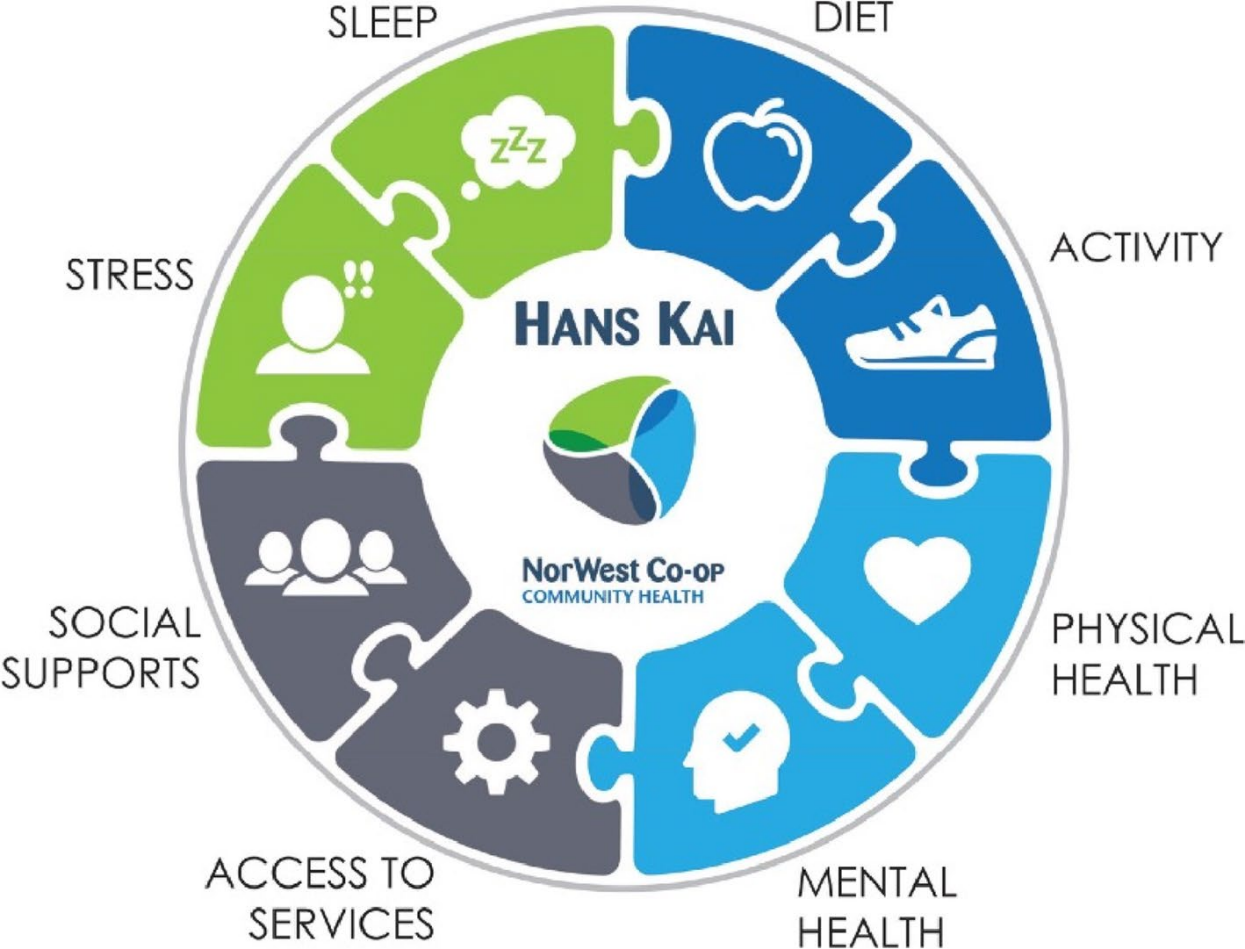
Hans Kai holistic model of wellness

Several features of this program distinguish it from other health promotion programs that are currently offered to Canadian adults, including the theoretical model behind it and its structure and content. Hans Kai is grounded in a holistic model of wellness [20] in which several social determinants of health at multiple levels of human ecology simultaneously interact to determine someone’s health status and overall wellbeing (see Fig. 1 for details). In line with this model, the program focuses on modifiable risk factors for poor physical health (e.g., diet, physical activity, noncommunicable diseases, access to health care services, and preventive care), as well as sleep, stress management, mental health, loneliness, and social support/connections.

The program is structured in two consecutive phases that are designed to promote participant retention and positive long-term health outcomes by improving health competence and decreasing social isolation and loneliness. First, Hans Kai participants attend a set of eight informational sessions, the Hans Kai Health School, that are delivered by healthcare professionals weekly in a group setting to develop the skills necessary to improve their health and wellbeing. The health school includes sessions on a variety of health-related topics, including the determinants of health; setting health goals and developing action plans; health indicators; healthy nutrition, grocery store shopping, and meal planning; physical activity; stress, coping, and health, including sleep; communicable and noncommunicable diseases, including modifiable risk factors; and access to healthcare services and preventive care.

After completing the health school, participants form Hans Kai groups and begin to meet regularly (at least once a month) at times, dates, and locations all determined by the group members, and independently of facilitators. Group size can vary from 5 to 30 members. To be classified as a Hans Kai group, at each meeting, the group members must conduct self-health checks, including blood pressure, blood glucose, and waist circumference measurements; they must also participate in some form of physical activity (e.g., Zumba or yoga), enjoy a healthy snack, and have time to socialize. The supplies for the health checks are provided by NorWest. The groups make all decisions collectively, and as they continue to meet, members may request to facilitators additional educational sessions not discussed in the core health school, or activities of interest of the group members (e.g., discussions on specific health topics, such as stroke or menopause, and cooking classes).

During the last health school session, each group chooses a group champion to lead the group and be the point of contact between the group and the Hans Kai staff. Hans Kai champions are not formally trained. They attend the health school together with all other Hans Kai participants and play the role of leaders following the health school graduation. During the Hans Kai group meeting period, the champions have several responsibilities, including organizing the group meetings and facilitating them in collaboration with the other group members; ensuring that the core Hans Kai activities are completed during each meeting and that the logbooks and forms are filled out; and contacting the Hans Kai staff if the toolkit needs restocking, to book a presentation, or for any other reasons.

The Hans Kai facilitators ensure that participants receive their Hans Kai education sessions as they prepare the group to meet on their own and learn how to complete the mandatory Hans Kai duties and fill out the participant logbooks.

The benefits of the Hans Kai program are achieved via health-related learning during the health school and peer support in their Hans Kai groups, which is meant to help participants improve their health and achieve their healthy lifestyle goals. The social connections that develop during Hans Kai and the peer support involved in being part of a Hans Kai group are thought to be the mechanisms by which the improvements in health and behavioral changes are achieved. The ultimate goal of the program is to reduce care burden on the healthcare system by promoting health-related self-efficacy and self-management in participants and ultimately preventing or reducing noncommunicable diseases, such as obesity, diabetes, cardiovascular diseases, as well as mood and anxiety disorders. The second portion of the Hans Kai program that involves regular group meetings can be adapted based on participants’ feedback and customized by participants to meet the needs of each specific peer-led group.

### Research objectives

The Hans Kai trial aims to address the three objectives listed below, one relating to the quantitative stand (trial objective 1) and two to the qualitative stand (trial objectives 2 and 3).

- *Objective 1.* To evaluate the effectiveness of the Hans Kai program in promoting the health and wellbeing of participants with respect to the following outcomes:
  - *1a.* Mental health (primary outcome).
  - *1b.* Social connections (secondary outcome 1), health-related knowledge and empowerment (i.e., self-efficacy and self-determination) (secondary outcome 2), and health-promoting behaviors (i.e., diet, alcohol consumption, tobacco use, physical activity, and sleep) (secondary outcomes 3 to 7).
  - *1c.* Cardiometabolic health outcomes (i.e., waist circumference, body weight, blood glucose, and blood pressure) (tertiary outcomes).
- *Objective 2.* To identify strengths and weaknesses of the Hans Kai program by investigating the experiences of those involved in facilitating and participating in the program.
- *Objective 3.* To identify barriers and facilitators to implementing the Hans Kai program from the perspectives of those who facilitate and participate in the program.

For the RCT component, it is hypothesized that during the trial, the Hans Kai participants will experience improvements in their physical health and wellbeing, including all mental and physical health outcomes measured. The qualitative component of the trial aims at answering the following two research questions: (1) what are the subjective experiences of those involved in facilitating and participating in Hans Kai regarding how and why the program works or does not work? (2) What factors facilitate or impede the implementation of Hans Kai from the perspectives of those who facilitate and participate in the program?

## Methods

RCTs are the gold standard for evaluating intervention effectiveness by assessing participant outcomes [21]. However, there is growing recognition that a broader mixed methods paradigmatic framework for evaluation research is warranted to generate patient-informed evidence that consider the complex perspectives and experiences of intervention participants and, therefore, are valuable for practice and policy development. In keeping with this innovative approach to evaluation research, the Hans Kai trial will follow a prospective mixed methods intervention design involving an RCT and an interpretative description study. The mixed methods intervention design is an approach to research in which the collection, analysis, and integration of both quantitative and qualitative data are embedded within an experimental quantitative research design [22]. The objective of gathering qualitative data within an experiment, along with the quantitative data on the outcome measures, is to gain an understanding of the personal, contextual experiences of the study participants.

In this mixed methods intervention study, quantitative data on the outcomes of interest will be collected along with qualitative data on the experiences of program participants and facilitators, and integrated within an intervention trial (i.e., RCT) to address the research objectives (see next section for details). The primary research design will be a 6-month RCT that will follow a 2-group parallel superiority design, with a 12–18-month observational follow-up including the intervention participants as well as participants who after completing the 6-month control period decide to participate in the intervention. The RCT and observational follow-up study will involve the collection, analysis, and interpretation of quantitative data (self-reports and objective indicators) on primary, secondary, and tertiary outcomes that will be gathered to evaluate the effectiveness of the Hans Kai program, relative to usual care, in promoting the health and wellbeing of Canadian adults with or without chronic conditions. The 12–18-month observational follow-up study will provide evidence on the long-term durability of any health changes observed during the 6-month RCT period.

To enrich the RCT results, the secondary qualitative strand of the study will be added during and after the intervention by embedding a combination of convergent and explanatory sequential designs into the RCT.

In the final phase of the trial, the quantitative results and qualitative findings will be integrated to determine how the qualitative findings enhance the RCT results by answering the following three questions: (1) how do the qualitative findings provide an enhanced understanding of the quantitative RCT results? (2) How do the experiences of the Hans Kai participants and facilitators help to explain the outcome results of the program? (3) How do the qualitative data gathered from the Hans Kai participants and facilitators regarding their involvement in Hans Kai help to explain the outcomes of the program?

### Randomized controlled trial

#### Study design

**Fig. 2:**
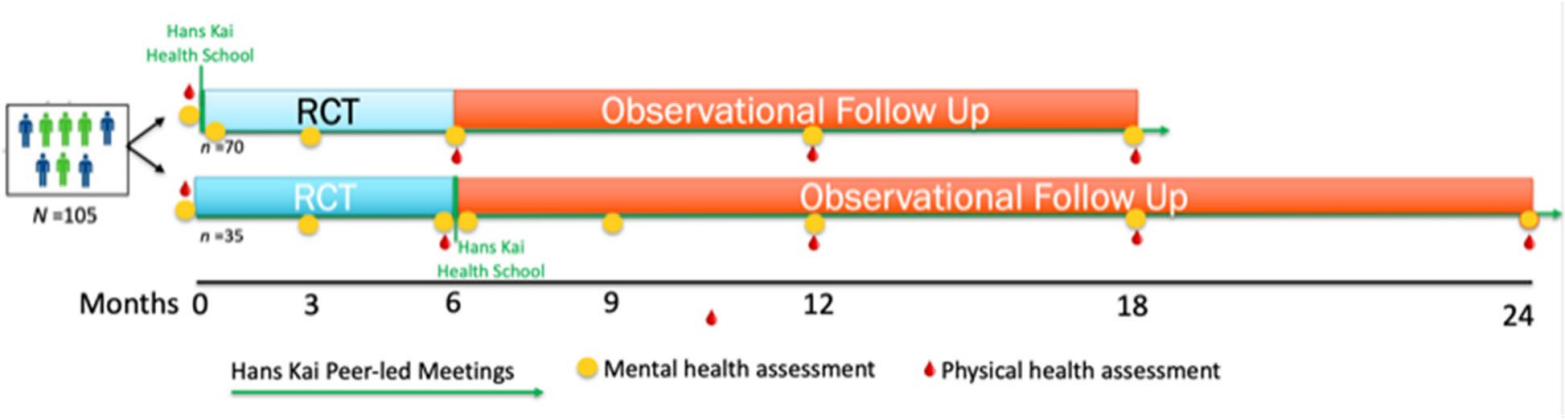
Hans Kai RCT design

The experimental component of the mixed methods RCT will follow a 6-month 2-group parallel superiority design, with a 12–18-month observational follow-up (see Fig. 2 for details). There will be a 2:1 allocation of participants to the intervention versus the control group. The control group will consist of 6 months of standard care that adults can access in the Canadian Province of Manitoba, including any services and programs that are offered at NorWest, except Hans Kai. At the end of the 6-month control period, the control group participants will be offered the opportunity to join Hans Kai and, once in the intervention, they will be followed for up to 18 months. The participants in the intervention group will be followed for a minimum of 6 months (RCT period) and up to 18 months; however, the randomized controlled portion of the study will last for the first 6 months, while the observational follow-up for them will last 12 months. After baseline, there will be a 2-month period of Hans Kai program implementation during which program participants will attend the Hans Kai Health School, prior to starting regular group meetings.

During the 6-month RCT, the participants who are randomly assigned to the intervention group will complete four visits, while the participants who are randomly assigned to the control group will complete three visits (see Supplementary Table 1 for the SPIRIT flow diagram). Following the intervention period (6 months), during the follow-up observational study, both groups will complete two visits 6 months apart. There will be no formal screening period as only participants who are eligible to enter the Hans Kai program will be invited to participate in the RCT.

#### Study setting

The study will be conducted primarily at NorWest, which is a multidisciplinary health clinic located in the city of Winnipeg (Manitoba, Canada) that engages the community in a variety of co-operative health-based initiatives. NorWest delivers community-based services and programs including primary healthcare, community development, counselling, support services, early learning, and childcare. Eligible individuals throughout Winnipeg can access services in the areas of family violence, immigrant and refugee services, substance abuse during pregnancy, nursing foot care, and Indigenous health. Hans Kai is an ongoing program offered at NorWest. Additional sites in the city of Winnipeg, including any Winnipeg Regional Health Authority (WRHA) site, will also be added as needed to reach the desired sample size of 105 participants (see next section for details). Based on a previous evaluation of Hans Kai [18], it is expected that approximately 85% of the trial participants (*n* = 90) will attend the Hans Kai program and complete the assessment visits at NorWest (15% dropout rate).

#### Recruitment

For the purposes of this trial, study participants will be recruited in the city of Winnipeg using multiple strategies. A short script describing the opportunity to participate in the Hans Kai trial was developed in a collaborative effort between the study team and the program staff. The script will be used to develop ad hoc products (e.g., posters, flyers, ads, and PowerPoint presentation slides) to advertise the study on social media (including, Facebook and Instagram), via postering, on local radio and television channels, via media releases, and by presenting this opportunity to different groups. Posters will be placed in multiple locations across the city of Winnipeg, including community health centers and resource centers, schools, churches, and cultural centers. Additional posting sites may be added as needed. Additionally, using a slideshow, the opportunity to join the Hans Kai trial will be presented to groups that may be interested in participating, such as seniors, mothers of young children, and groups supported by community centers in the city of Winnipeg, which will be approached at the sites.

#### Participants

Participants may enter the trial if all the following apply: (i) age 18 years or older; (ii) any gender; (iii) willingness and ability to give informed consent for participation in the trial; (iv) ability to speak and read English at a grade 6 level; (v) self-reported motivation to making lifestyle changes that can positively impact someone’s health; (vi) stable health status that allow participating in the program activities, including performing light exercise (as determined by the participant and Hans Kai staff); and (vii) ability and willingness to comply with all trial requirements (as determined by the research team). Participants may not enter the trial if any of the following apply: (i) age under 18 years; (ii) cognitive impairment that prevents the person from providing informed consent or participating in the program activities (as determined by the study staff); (iii) existing relationship with the research team, such as supervisory or familial relationship; (iv) frailty that prevents the person from participating in group activities or exercise (as determined by a healthcare professional); (v) participation in another research trial in the past 12 weeks; (vi) instable health or serious illness, such as, dementia or a terminal illness, or recent significant medical diagnosis; and (vii) inability to comply with all trial requirements.

A research team member with extensive experience in conducting mixed methods research will screen potential participants, enrol eligible participants in the trial, and collect written informed consent from all trial participants. If required, following appropriate training, research assistants will also be involved in this process to support the research team.

#### Sample size

The primary analysis will be performed based on the pre- to post-intervention changes in the primary outcome, the Mental Health Continuum Short Form (MHC-SF) [23], comparing the mean change scores between the intervention and control participants, with a nested design to account for participant group membership in Hans Kai [24]. Based on data from previous studies [4, 24] and the RCT study design, we expect the changes from baseline MHC-SF scores within each subject group to be approximately normally distributed with a standard deviation of 5. The average within-group pre-post changes in MHC-SF scores in the Hans Kai prospective cohort study were + 6.5 in 34 participants at 6 months [18]. With a planned sample size that accounts for dropouts of 90 adults, with 60 intervention participants and 30 control participants, we would have a power of 80% to detect a true difference in the mean response of intervention and control participants of − 3.1 or 3.1 at the end of the 6-month RCT component [25]. This scenario assumes that the outcomes of each participant will be independent of the Hans Kai peer group they join. However, since part of the Hans Kai program includes forming groups, a very conservative power calculation considers the “effective” sample size for the Hans Kai program to be equal to the number of groups rather than the number of participants. We anticipate that 60 intervention participants will form 10 independent groups of six participants per group. In this very conservative scenario, with 10 groups in the intervention arm and 30 control participants, we would be able to detect a true difference in the mean scores of intervention and control participants of − 5.2 or 5.2 with 80% power. We believe that the actual study power will fall somewhere between the two scenarios presented here. With a starting sample size of 105 adults and an estimated 15% dropout rate during the study period [18], we expect to generate a minimum sample of approximately 90 participants at the 18-month follow-up.

#### Randomization

Following the eligibility screening and the baseline assessment visit, participants will be randomly allocated to either the intervention group or the control group using a computer-generated random sequence. After screening and enrollment, once participants are determined to be eligible to participate in the clinical trial, a research team member will press a randomize function within the REDCap secure system. Participants will be assigned with a 2:1 ratio of intervention to control groups. To ensure allocation concealment and minimize bias, randomization will be performed by a third-party biostatistician not affiliated with the research team. The randomization sequence will be uploaded into the University of Manitoba Research Electronic Data Capture (REDCap) system randomization module [26, 27].

#### Blinding

Blinding of participants and study staff is not possible given the nature of the intervention, but the study statistician and the entire data management team will be blinded to allocation during analysis, making this a prospective randomized open blinded end-point study [28].

#### Intervention

In this trial, there will be two groups, an intervention group (*n* = 70) that will immediately enrol in the Hans Kai program and a control group (*n* = 35) that will access standard care available in the Canadian Province of Manitoba, including any services and programs offered at NorWest, except Hans Kai. There will be no special criteria for discontinuing or modifying allocated interventions, except in cases involving worsening of pre-existing health conditions that may either put participants at risk if they continue the program or hinder their participation in the program. The program that will be the intervention in this trial is the Hans Kai program offered at NorWest, with the only modifications being the trial assessment visits described below. The minimum level of compliance to the intervention is 80%; to remain in the trial, study participants must attend a minimum of six health school sessions.

Participants in the control group will remain as close to a “typical” community member as possible as they will be able to receive any other health programming normally available to them in Winnipeg, except the Hans Kai program. Standard of care will be made available to all members of the control group in compliance with the healthcare rights of Canadians and Manitobans holding a Manitoba health card. NorWest staff will provide support to the community members without a Manitoba health cards in obtaining one. The only deviation from standard care in the control group will be the baseline, 3-month, and 6-month visits described below during which the control participants will complete questionnaires and undergo physical assessments.

#### Outcomes

All study outcomes will be measured pre- and post-intervention at multiple timepoints and will be assessed using a multi-method approach involving subjective self-report measures and objective indicators (see Table 1 for details). The primary objective of this RCT is to measure the impact of the Hans Kai program on participant mental health using the MHC-SF [24] by comparing the MHC-SF scores obtained by the intervention group with those of the control group. The MHC-SF is a 14-item standardized self-report questionnaire that assesses emotional, social, and psychological wellbeing of respondents by investigating the regularity with which they experience symptoms of positive mental health. This scale shows high internal consistency (*α* = and moderate test–retest reliability, as well as good convergent and discriminant validity when it is used to measure mental wellbeing in Western contexts [23].

**Table 1:** Quantitative objectives, outcome measures, and timepoints of assessment

Objectives	Outcome measures	Timepoints of assessment
*Primary objective:*		
To compare the effects of the Hans Kai program versus standard care on mental health	Mental Health Continuum Short Form (MHC-SF)	The MHC-SF will be completed at each assessment visit
*Secondary objectives:*		
1) To compare the effects of the Hans Kai program versus standard care on social connections	1) Revised UCLA Loneliness Scale	The UCLA Loneliness Scale will be administered at baseline and then every 6 months of the Hans Kai program or standard care control
2) To compare the effects of the Hans Kai program versus standard care on health-related knowledge and empowerment (i.e., self-efficacy and self-determination)	2) Perceived Health Competence Scale (PHCS)	The PHCS will be administered at baseline and then every 6 months of the Hans Kai program or standard care control
3) To compare the effects of the Hans Kai program versus standard care on health-promoting behaviors (i.e., diet, alcohol consumption, tobacco use, physical activity, and sleep)	3a) Modified version of the Healthy Eating Assessment 3b) Pittsburgh Sleep Quality Index (PSQI) 3c) Fitbit	All self-report measures will be administered at baseline and then every 6 months of the Hans Kai program or standard care control Participants will be asked to wear a Fitbit for a total of seven days at baseline and then every 6 months of the Hans Kai program or standard care control
*Tertiary objective:*		
To compare the effects of the Hans Kai program versus standard care on clinical measures of cardiometabolic health	Clinical exams will be performed by a registered nurse	Waist circumference, blood pressure, fasting blood sugar, and heart rate will be measured at baseline and then every 6 months of the Hans Kai program or standard care control

The secondary objectives of this RCT involve investigating the impact of the Hans Kai program on social connections, health-related knowledge and empowerment (i.e., self-efficacy and self-determination), and health-promoting behaviors (i.e., diet, alcohol consumption, tobacco use, physical activity, and sleep). To this end, a set of self-report questionnaires as well as objective measures will be used to assess changes in these outcomes over time (see Table 1 for details).

Feelings of loneliness and social isolation (secondary objective 1) will be measured using the revised UCLA Loneliness Scale [29], a 20-item scale designed to measure one’s subjective feelings of loneliness and social isolation. This scale is highly reliable (internal consistency *α* coefficient = 0.89–0.94 and test–retest reliability over a 1-year period *r* = 0.73) and valid in assessing self-reported loneliness and social isolation in adults.

Health-related knowledge and empowerment in the form of self-efficacy and self-determination (secondary objective 2) will be measured using the Perceived Health Competence Scale (PHCS) [30]. This is a standardized measure of general health management self-efficacy beliefs designed to assess respondents’ self-perceived ability to accomplish health-related goals and manage their health positively. The scale has good internal consistency (0.82-0.90) and test–retest reliability (0.82–0.60), as well as demonstrated construct validity.

Change in health-related behaviors (secondary objectives 3 to 5) will be assessed using a combination of objective indicators and subjective measures. Changes in nutritional behavior (diet), alcohol consumption, and tobacco use will be measured using a modified version of the Healthy Eating Assessment [31] that was specifically created for the purpose of this trial. The adapted self-report scale includes 22 items divided into two main sections: a first section that includes 18 items evaluating respondents’ eating habits, and a second section including four items that assess respondents’ alcohol consumption and tobacco use.

The standardized self-report questionnaire Pittsburgh Sleep Quality Index (PSQI) [32] will be used to subjectively measure sleep quality, habits, and patterns subjectively. This scale includes nine items that assess seven aspects of sleep: (1) subjective sleep quality, (2) sleep latency, (3) sleep duration, (4) habitual sleep efficiency, (5) sleep disturbances, (6) use of sleeping medications, and (7) daytime dysfunction over the last month. Scoring varies across items; however, an overall score can be calculated by summing the seven component scores, yielding a Global PSQI score ranging from 0 to 45, with higher scores indicating poorer sleep quality (secondary outcome 6). The PSQI has shown acceptable internal homogeneity, consistency (test–retest reliability), and validity. Additionally, a global PSQI score greater than 5 yield a diagnostic sensitivity of 89.6% and specificity of 86.5% (*k* = 0.75, *p* < 0.001) in distinguishing good and poor sleepers.

Sleep quality (Fitbit sleep score) and length (sleep time) will also be measured objectively using data provided by a Fitbits that participants will wear for 7 days at each assessment point [33]. The average of these seven measurements will be used.

Fitbits will also be used to measure behavioral changes in terms of physical activity (secondary outcome 7) [34, 35]. Specifically, at each assessment point, daily step count and moderate-to-vigorous physical activity will be recorded over a 7-day period, and the average of these seven measurements will be used as an indicator of participants’ level of physical activity.

The tertiary objective of this RCT is to investigate the impact of the Hans Kai program on clinical cardiometabolic health indicators, including blood glucose, waist circumference, body weight, and blood pressure. These indicators will be assessed objectively by a registered nurse who every 6 months will perform a clinical exam of study participants.

#### Procedure

At the baseline assessment, all participants (both the intervention and control group) will undergo a clinical exam that will be performed by a trained research team member, who will measure participants’ blood glucose levels, blood pressure, weight, and waist circumference according to provincial health standard protocols. All clinical measurements will be taken three times, except blood glucose, which will be measured only once using a glucometer that tests capillary blood samples taken by finger prick sampling. Blood pressure will be measured in triplicate following 10 min of acclimatization to the measurement room, with a 2-min break between measurements. The average of the three assessments will be used as measure of blood pressure. Following the clinical assessment, participants will be asked to complete all self-report measures, including a sociodemographic questionnaire, the MHC-SF, the Revised UCLA Loneliness Scale, the PHCS, the Adapted Healthy Eating Assessment, and the PSQI. Fitbits will also be used to objectively assess changes in participants’ physical activity as well as sleep quality and patterns. Participants will be shown by the study staff how to wear the Fitbit and will be given basic instructions on its use. Study participants will be asked to wear the Fitbit for 7 complete days on the assessment week, including when they are asleep so that their sleep quality and patterns can be recorded.

**Fig. 3:**
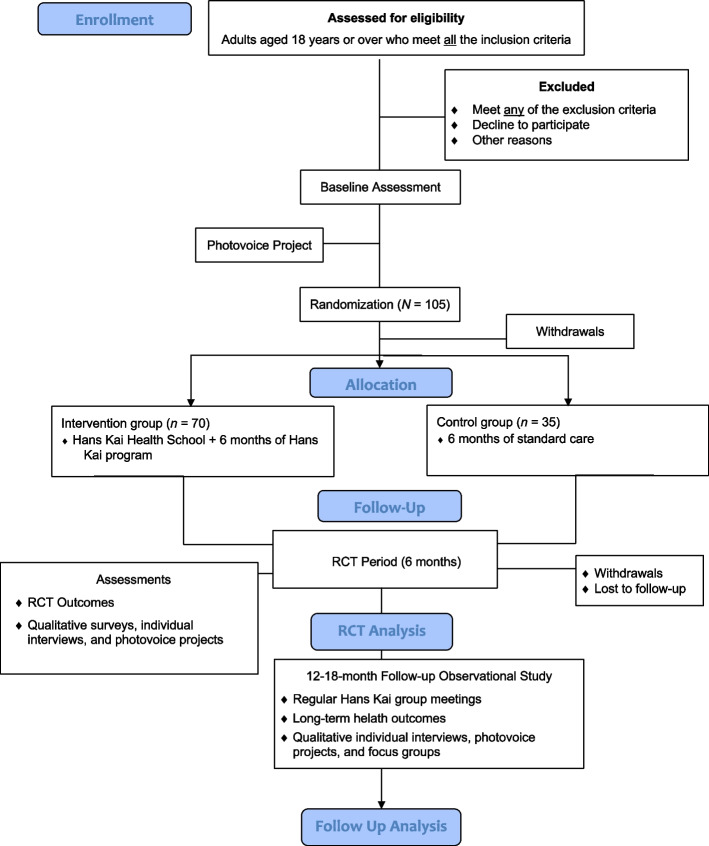
Consort flow diagram

**Fig. 4:**
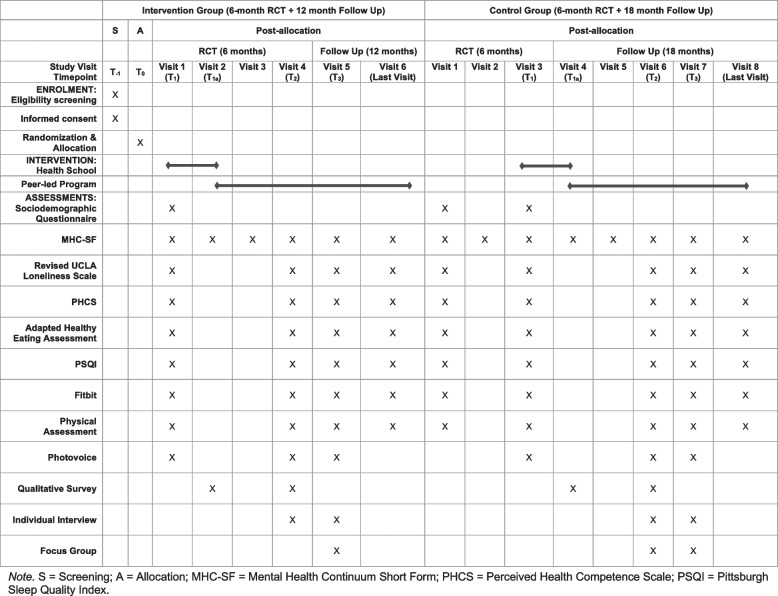
SPIRIT flowchart of the schedule of enrolment, intervention, and assessments

Following the baseline assessment, study participants will be randomly assigned to either the intervention group or the control group (see Fig. 3 for Consort flow diagram) [36]. After random allocation to either trial condition, participants in the intervention group will immediately enter the Hans Kai program, while participants in the control group will be exposed to 6 months of standard care. Participants who are randomized to the control group will receive $100 CAD (made out in two payments of $50 CAD, one at the 3-month visit and one at 6-month visit). This remuneration for participants who are randomly assigned to the control group instead of the intervention group will be used as a strategy to improve adherence to the intervention protocol. We will also monitor adherence by tracking attendance to the health school and trial visits. See Fig. 4 for the SPIRIT flowchart of the schedule of enrollment, intervention, and assessments.

##### The 6-month RCT

During the 6-month RCT period, the intervention group will complete the health school (2 months) and start regular Hans Kai group meetings (6 months). During this period, the intervention group participants will complete the MHC-SF at the end of the health school and 3 months after the end of the health school, as well as all physical and mental health assessments 6 months after the end of the health school. During the 6-month RCT period, the control group will have access to standard care and will complete the MHCF-SF (3 months after enrollment) as well as all physical and mental health assessments (6 months after enrollment). In both groups, the primary outcome mental health, measured via the MHC-SF, will be assessed at baseline as well as at the 3-month and 6-month timepoints.

##### The follow-up observational study

The follow-up observational study will last a total of 18 months for the control group and 12 months for the intervention group. The participants coming from the control group will first complete the Hans Kai health school (2 months) and then in the first 6 months of the follow-up study, they will complete the same visits that were completed by the intervention group during the 6-month RCT period, followed by two full assessments, including all physical and mental health measures that will be performed 6 months apart (12 additional follow-up months). During the follow-up study, the participants coming from the intervention group will complete two full assessments, including all physical and mental health measures that will be performed 6 months apart.

#### Statistical analysis

Preliminary analysis will involve computing descriptive statistics for all quantitative variables. For continuous variables, means, standard deviations, and quartiles will be estimated, while categorical variables will be summarized with counts and percentages in each category. Summaries will be performed by group and by assessment, as well as for the entire study group. Primary data analysis of the RCT portion will involve comparing the mean values of the within-subject changes in outcome variables from baseline to 6 months between the treatment and control groups. Secondary data analysis will include a test of the interaction between time in the Hans Kai program and the outcome measurements in all participants from 0 to 20 months. The primary analysis will be conducted at the 0.05 level of significance. In general, test results will be described as significant if their *p*-values are less than 0.05 without adjustments for multiple inference. Multiple imputation will be used to deal with missing data.

### Interpretive description

#### The interpretive description methodology

The qualitative component of the Hans Kai trial will follow an interpretive description design that will be implemented during and after the intervention by embedding a combination of convergent and explanatory sequential designs into the RCT. Interpretive description is a flexible qualitative research methodology for conducting rigorous qualitative research that generates knowledge relevant for clinical practice in the applied health disciplines [37, 38]. The overarching aim of interpretive description is to provide practical evidence on subjective experiences valuable in informing future decisions in health-related settings that impact the lives of different populations. In the context of the Hans Kai trial, this qualitative research methodology will be used to generate new knowledge on the subjective experiences of study participants whose findings are meaningful and applicable to clinical practice.

In this research, the interpretive description methodology will be used to investigate the experiences of Hans Kai program participants and facilitators to identify the main strengths and limitations of Hans Kai, uncover potential implementation issues, and elucidate the mechanisms through which the program works. This qualitative component of the trial will involve a multi-method approach in which multiple qualitative data collection methods will be used to reach a deeper and more nuanced understanding of the program strengths, how the program works, and for which people it is more effective in promoting health and wellbeing. Adaptable components of the program will also be investigated and modified according to the feedback provided by the trial participants.

#### Research objectives

##### The convergent component

The qualitative data that will be collected during the intervention component of the study will address process questions. This component of the study aims to (1) gain an understanding of the experiences of the program participants and facilitators with the program; (2) identify the strengths and weaknesses of the program, as well as the barriers and facilitators to implementing the program from the perspectives of the program participants and facilitators; and (3) receive feedback from program participants and facilitators to revise the modifiable aspects of the program. This data will complement the RCT outcome data.

##### The explanatory sequential component

Case-selection variant

The qualitative data that will be collected after the intervention component of the study will aid in interpreting the RCT outcome results and explaining why the intervention worked or did not work, or why it worked for certain participants but not others. Specifically, the aims of this qualitative strand are (1) to understand why the outcomes occurred; (2) to explain variations in outcome results across program participants; (3) to elucidate the mechanisms through which the program works; (4) to assess how context may influence the program effectiveness; and (5) to receive feedback from program participants and facilitators to revise the modifiable aspects of the program.

##### Participant selection

In keeping with the explanatory sequential research design—case-selection variant, to address the stated research objectives, three subsamples of program participants will be selected using purposeful sampling based on the results of the first outcome assessments (Time 2: 6-month follow-up). These three groups will include the following:

- *Group 1:* 8–10 program participants who experienced improvements of 25% or more in all or most outcomes measured between Time 1 (baseline) and Time 2 (6-month follow-up).
- *Group 2:* 8–10 program participants who between Time 1 and Time 2 experienced improvements of 25% or more in some outcomes *but* no change or any deterioration in other outcomes.
- *Group 3:* 8–10 program participants who experienced no change or any deterioration in all or most outcomes measured between Time 1 and Time 2.

Examining the perspectives of program participants who experience diverse program outcomes will aid in interpreting the results of the experimental RCT component by allowing to reach a deeper and richer understanding of the mechanisms through which the program works in promoting participants’ health and wellbeing, for whom, and under what circumstances.

#### Qualitative research activities

##### The convergent component

A short survey including a set of open-ended questions will be administered to all Hans Kai participants at Time 1a (at the termination of the health school) and Time 2 (6-month follow-up) to address the stated research objectives. The survey will include eight open-ended questions investigating what motivated study participants to join Hans Kai and what they hope to gain by participating in the program; their perceived strengths and weaknesses of the program; and the barriers and facilitators they experienced during the program. A similar survey including five open-ended questions will also be administered to all Hans Kai facilitators at the beginning of the RCT (Time 1: baseline).

##### The explanatory sequential component

Individual face-to-face in-depth semi-structured interviews will be conducted with a sample of 24–30 Hans Kai participants following the Time 2 outcome assessments (6-month follow-up) and then again at Time 3 (12-month follow-up or 1 year after participants enrolled in the program). The 24–30 participants will be purposefully selected based on the results of the Time 2 outcome assessments (see previous section for details) so that this subsample of study participants will include a combination of adults who experienced improvements, no changes, or deterioration in the health outcomes measured in the experimental component of the RCT to evaluate program effectiveness. The individual interviews will involve a set of open-ended questions that aim to investigate study participants’ subjective experiences in the Hans Kai program. Participants’ accounts generated through these interviews will aid in the interpretation of the RCT results. The same interview guide will also be used to conduct a focus group (involving 5–8 participants each) with all participants who will still be enrolled in Hans Kai 1 year after joining the program at Time 3. Additionally, at Time 1, 2, and 3 a purposefully selected sample of 30 program participants (10 at each timepoint) will be invited to participate in a photovoice project involving taking pictures regarding their subjective experiences of being and Hans Kai participant. Once the photovoice projects are complete, study participants will be invited to create a storytelling video describing their health journey before and during Hans Kai by integrating their pictures into a video. At Time 2 and Time 3, the photovoice participants will be selected using the same criterion used to select the individual interview participants to ensure including participants who experience diversified program outcomes.

#### Data analysis

All narrative and pictorial data collected during the qualitative research activities will be included in the qualitative analysis. Following recommendations by Charmaz [39, 40], in order to explore nuances of meaning and generate an understanding of study participants’ perspectives that accurately reflects their subjective experiences, two research team members will use a constant comparative method to analyze all qualitative data (narrative and pictorial). This method will entail engaging in both data collection and analysis simultaneously by conducting data collection and analysis in a parallel, iterative process, with coding of qualitative materials starting while the research activities are still in progress. This procedure will allow learning from the emerging data about participants’ subjective experiences since the beginning of the research process. This data will then be used to develop progressively more focused interview protocols.

Throughout the research process, informal analytic notes, commonly referred to as memos, will also be developed [39, 40]. Memo-writing represents a central tool in qualitative coding because it prompts us to analyze the data and codes early in the research process. To engage in memo-writing, we will stop and think about our participants, their experiences, and their meanings, as well as about our own experiences and preconceived assumptions through the process of reflexivity. We will write our memos for personal use in an informal language and in a spontaneous fashion, and we will create a “memo bank” to store our memos. Specifically, the interviewer will write extensive narrative descriptions that will include (i) fieldnotes about the data collection activities; (ii) memos about the data collection and analysis process; and (iii) reflexive notes about the interviewer’s personal assumptions [41]. In these personal, reflexive memos, which will be written at the conclusion of each interview, the interviewer will use reflexivity to acknowledge how their background, experiences, values, and beliefs may have impacted how the study was being conducted.

Transcribed interviews, group discussions, and survey responses, as well as photos and investigator memos, will be included in the analysis. In grounded theory, data analysis consists of studying the early data and begin to separate, sort, and synthesize these data through qualitative coding [39, 40]. Qualitative coding means naming segments of data with a label that simultaneously categorizes, summarizes, and accounts for each piece of data. Grounded theory coding requires us to stop and ask analytic questions of the data we have gathered to further our understanding of participants’ experiences of the Hans Kai program, and to help us direct subsequent data-gathering toward the analytic issues we are defining. In coding the qualitative materials, we will follow Charmaz’s recommendations to conduct coding in two main phases, including initial coding and focused coding, and by performing both an intra- and inter-participant thematic analysis [see 36 and 37 for more details].

#### Trustworthiness

In qualitative inquiry, trustworthiness of the data refers to the degree to which the study findings are supported by evidence and can be trusted as accurate reflections of participants’ perspectives on the research topic [42]. In the Hans Kai trial, the trustworthiness of the data will be ensured by implementing a series of strategies that endeavor the credibility, transferability, dependability, and confirmability of the data.

Credibility will be enhanced implementing methodological triangulation [42, 43]. Using multiple methodologies or data sources (including surveys, individual interviews, focus group discussions, and photovoice projects) to take a multifaceted view of study participants’ perspectives and subjective experiences will add rigor, depth, complexity, and richness to the research study. The findings of this qualitative component of the trial will provide essential context and meaning to the interpretation of the RCT results. Further strategies that will promote credibility involve using the qualitative data analysis software Nvivo for data management and analysis; confirming researcher understandings and interpretations of participants’ accounts by summarizing the main points of the individual interviews, photovoice projects, and focus group discussions to participants at the conclusion of each research activity; and engaging in extensive memo writing to support reflection on the emerging data and document procedures, insights, and analytic decision-making. Additionally, maximum variation sampling will be used to collect data from the widest range of perspectives possible and construct a holistic understanding of the factors that may impact the implementation and effectiveness of the Hans Kai program [44].

Transferability of findings to other contexts (settings and populations) will be endeavored through a “rich, thick description” of study participants and their multiple perspectives and subjective experiences that will help provide a clear description of the context in which the study findings were generated so that others will be able to determine whether the application of these findings to other populations is appropriate, given the characteristics of the two groups [42, 43]. Strategies used to increase the dependability of the qualitative findings will include continuity and trust in the relationship between the researchers and the study participants, and involvement of a researcher with qualitative research expertise who will assist in all steps of the research process and aid in ensuring that these conform to the standards necessary to provide an accurate representation of participants’ perspectives and experiences [43]. This researcher, together with other team members with content expertise, will also be involved in peer debriefing and audit trial.

Confirmability will be ensured by using participants’ direct quotes as evidence of the study findings [42]. Reflexivity will also be used throughout the research process to clarify investigator personal values, attitudes, assumptions, and worldviews that may impact the analytic decision-making process. In this regard, fieldnotes, memos, and reflexive notes will be used as part of the research team discussions to help the investigators take a reflexive stance by examining and clarifying how their past life experiences, worldviews, and assumptions may affect the research process [41]. Great attention will be paid to preventing these assumptions and biases from affecting the data analysis process.

### Integrating the quantitative and qualitative findings

In the final phase of the trial, the qualitative themes reflecting the unique experiences of the Hans Kai participants and facilitators will be used to explain the quantitative numeric RTC results on the effectiveness of Hans Kai by comparing the two sets of data. Specifically, after analyzing the quantitative and qualitative data, a joint display showing the relationship between the intervention benefits and participant experiences will be developed and used to integrate the two sets of data [22]. For each trial participant who completed at least one qualitative research activity, improvements, with magnitudes, as well as no changes and deteriorations, with magnitudes in all outcomes of interests will be listed in the joint display and connected to the themes reflecting each participant’s unique experience. Using the RCT outcomes, we will create typologies that illustrate different intervention benefits (e.g., benefits in physical health only, benefits in physical and mental health, or benefits in mental health only) and, for each category in the typology, we will interpret the RCT results by comparing the changes in the outcomes of interest with differing participant experiences. This will lead to additional insights into the effectiveness of the Hans Kai program. The qualitative themes reflecting facilitators’ experiences will be kept separate from the RCT results.

### Data management

All quantitative data, including self-report and objective measures, as well as the survey data will be collected, managed, and stored using REDCap electronic data capture tools [26, 27]. REDCap is a secure, web-based software platform designed to support online and offline data capture for research studies and operations by providing: (i) an intuitive interface for validated data capture; (ii) audit trails for tracking data manipulation and export procedures; (iii) automated export procedures for seamless data downloads to common statistical packages; and (iv) procedures for data integration and interoperability with external sources (see http://project-redcap.org for details). This secure web-based application for building and managing online surveys and databases will be used to virtually collect all study data, including informed consents. The REDCap Consortium, a vast support network of collaborators, is composed of thousands of active institutional partners in over one hundred countries. All data will be entered into REDCap by trial participants or a research team member at the time of completing the assessments.

Individual interview, photovoice interviews, and focus group discussions will be audio recorded, transcribed verbatim by a professional transcriptionist or a research team member, de-identified, and imported into the software NVivo (version 12) for data management and analysis. Survey responses will first be entered in REDCap by study participants and then imported in Nvivo for inclusion in the data analysis process. All qualitative data will be collected by a research team member with extensive experience in qualitative research.

### Data monitoring

Given the nature of the Hans Kai program, it is very unlikely that any adverse events (AEs) will be related to the trial. However, all AEs occurring during the trial that are observed by the research team or reported by participants will be recorded on participants’ case report forms (CRFs), whether or not they are attributed to the trial intervention. The following information will be recorded: description, date of onset and end date, severity, and assessment of relatedness to the trial intervention. Follow-up information will also be collected as necessary. The severity of the AEs will be assessed on the following scale: 1 = mild, 2 = moderate, 3 = severe. AEs considered related to the trial intervention as judged by a qualified physician (general practitioner) at the NorWest Clinic will be followed until either resolution or the event is considered stable. Given the low risk involved in participating in the Hans Kai trial, a Data and Safety Monitoring Board (DSMB) will not be assembled, and no planned stopping guidelines were developed. The sponsoring institution (University of Manitoba) may audit the study data as part of the ongoing research audit they conduct for trials that they sponsor. Regular monitoring will be performed according to good clinical practice by the Principal Investigator (PI) or a delegate. A subset of all data will be evaluated for compliance with the protocol and accuracy in relation to source documents. All primary outcome measurements for each participant will be verified for accuracy.

### Ethical considerations

Digitally written informed consent will be collected in REDCap from all study participants before performing any research activity by a research team member with extensive experience in mixed methods research. To ensure eligibility to participate in the trial, the researcher will review the inclusion and exclusion criteria with potential participants prior to beginning the informed consent process. Prospective participants will be asked to personally sign and date the latest approved version of the informed consent forms before any trial procedure is performed. During the informed consent process, study participants will be informed of their right to withdraw from the study at any time without dropping out of the Hans Kai program or impacts on the other services they may be receiving at NorWest. Study participants who voluntarily withdraw from the study (dropouts) will be invited to participate in a short exit interview involving questions that investigate their subjective experiences in Hans Kai and the reasons for leaving the study and perhaps also the program.

The research team will ensure that the participants’ anonymity and confidentiality is protected at all times during the study period. All study participants will be identified only by a participant ID number on all trial documents and in any electronic database (e.g., REDCap). All study documents will be stored securely and will be accessible only by the research team. The trial results will be disseminated through a variety of strategies, including academic publications, research reports, infographics, media releases, and community events. Trial participants’ anonymity and confidentiality will be protected during these activities by removing all identifiable information from the knowledge dissemination products.

The trial will comply with The Personal Health Information Act (PHIA) [45] and The Freedom of Information and Protection of Privacy Act (FIPPA) [46] of Manitoba. Ethics approval was granted by an institutional Research Ethics Board (REB) (Winnipeg, Manitoba, Canada), and by Shared Health, the provincial health organization that integrates and coordinates the planning of patient-centered care across Manitoba. Trial progress reports will be sent to the institutional REB for ethics approval on an annual basis. Any deviations from the research protocol approved by these entities will be described in amendments that will be submitted for approval prior to implementing the deviations.

### Knowledge translation

The quantitative and qualitative trial results as well as the mixed methods trial findings will be shared at national and international conferences, through academic publications and community events, and on the Norwest website to share what is learned throughout the trial with participants, healthcare professionals, and the public.

## Discussion

Cancer, diabetes, cardiovascular disease (heart disease and stroke), and lung disease are the leading causes of preventable death and disability in Canada [47]. Canadians can dramatically reduce their risk of experiencing these chronic diseases—noncommunicable diseases—by implementing everyday lifestyle choices and health-promoting habits relating to their diet, physical activity, and stress management that reduce their risk of experiencing these conditions. It is therefore critical to evaluate if Canadian adults benefit from the health promotion intervention Hans Kai more than standard service provision. The results of this trial will provide important insight into the effectiveness of the Hans Kai program in promoting the health and wellbeing of Canadians with and without chronic health conditions. A broad range of outcomes that could potentially be affected by Hans Kai will be measured, and the results of these assessments are anticipated to inform health promotion policy development in Manitoba.

The qualitative component of the Hans Kai trial will enhance the interpretability of the quantitative RCT results through data triangulation [48]. The inclusion of the interpretive description study will serve three additional purposes. First, it will provide insight into the subjective experiences of study participants with the Hans Kai program and how their personal characteristics and circumstances may have shaped those experiences. This set of data will help us better understand whether the Hans Kai program works better for certain persons in specific contexts rather than others and why. Understanding these experiences is highly relevant to the implementation of health promotion interventions in diverse contexts to ensure that all participants are engaged and motivated to actively take part in these programs. Additionally, the qualitative findings will provide valuable information about the outcomes of health promotion interventions that are important to participants. By targeting these outcomes, health promotion programs are more likely to retain participants and truly have an impact on their health and wellbeing. Finally, the qualitative component of the Hans Kai trial will shed light on the mechanisms through which the program positively affects the health and wellbeing of its participants. This knowledge may be used to not only improve the Hans Kai program, but also develop new, more effective community-based health promotion interventions that involve the same mechanisms of action.

### Supplementary Information

**Additional file 1.** Spirit Checklist.

## Data Availability

The quantitative datasets analyzed during the current study and statistical codes are available from the corresponding author on reasonable request, as is the full protocol. The participant information materials and informed consent form are also available from the corresponding author on request.

## Abbreviations

AEs: Adverse Events
CRFs: Case Report Forms
DSMB: Data and Safety Monitoring Board
FIPPA: Freedom of Information and Protection of Privacy Act
MHC-SF: Mental Health Continuum Short Form
NorWest: NorWest Co-op Community Health
PHCS: Perceived Health Competence Scale
PHIA: Personal Health Information Act
PI: Principal Investigator
PI: Principal Investigator
PSQI: Pittsburg Sleep Quality Index
RCT: Randomized Controlled Trial
REB: Research Ethics Board
REDCap: Research Electronic Data Capture
WRHA: Winnipeg Regional Health Authority
